# New-Onset Type 1 and Type 2 Diabetes Among Korean Youths During the COVID-19 Pandemic

**DOI:** 10.1001/jamapediatrics.2024.5068

**Published:** 2024-12-09

**Authors:** Da Hye Lee, Hwa Young Kim, Ji Young Park, Jaehyun Kim, Jae Hyeon Park

**Affiliations:** 1 Department of Pediatrics, Chung-Ang University Hospital, Seoul, Republic of Korea; 2 Department of Pediatrics, Seoul National University Bundang Hospital, Seongnam, Republic of Korea; 3 Department of Pediatrics, College of Medicine, Seoul National University, Seoul, Republic of Korea; 4 Department of Pediatrics, Korea University Ansan Hospital, Ansan, Republic of Korea; 5 Department of Laboratory Medicine, Seoul National University Hospital, Seoul, Republic of Korea

## Abstract

**Importance:**

Whether COVID-19 contributes to youth-onset diabetes is controversial, and research in Asia is lacking.

**Objective:**

To explore the incidence and severity of diabetes among youths during the COVID-19 pandemic in South Korea.

**Design, Setting, and Participants:**

This cohort study used claims data for January 1, 2017, through February 28, 2022, from the National Health Insurance Service database in South Korea. The incidence of type 1 diabetes (T1D) and type 2 diabetes (T2D) in patients younger than 20 years during the pandemic was analyzed and compared with that during the prepandemic period. The study included incident cases of T1D identified by at least 2 diagnosis codes with at least 2 insulin prescriptions within 1 year and T2D identified by at least 2 diagnosis codes with at least 2 prescriptions of diabetes medication within 1 year. Analyses were performed between January 29 and September 2, 2024.

**Exposures:**

COVID-19 pandemic and SARS-CoV-2 infection.

**Main Outcomes and Measures:**

The primary outcome was incidence of T1D and T2D, and secondary outcomes included the rate of diabetic ketoacidosis (DKA) and association of new-onset diabetes with SARS-CoV-2 positivity.

**Results:**

The study included 2599 patients with T1D (mean [SD] age, 12.0 [4.8] years; 1235 [47.5%] male) and 11 040 patients with T2D (mean [SD] age, 16.0 [2.8] years; 6861 [62.1%] male). During the pandemic, the incidence rate ratios were 1.19 (95% CI, 1.10-1.29) for T1D and 1.41 (95% CI, 1.36-1.46) for T2D. The incidence rate of DKA at diagnosis increased during the first pandemic year compared with the prepandemic period (T1D, 42.8% [95% CI, 38.5%-47.0%] vs 31.3% [95% CI, 29.0%-33.7%], respectively; T2D, 6.0% [95% CI, 5.0%-7.1%] vs 2.9% [95% CI, 2.5%-3.3%], respectively) but returned to prepandemic levels in the second pandemic year (T1D, 34.5% [95% CI, 30.6%-38.5%]; T2D, 3.2% [95% CI, 2.6%-3.9%]). The hazard ratio for new-onset diabetes associated with SARS-CoV-2 positivity was 0.44 (95% CI, 0.17-1.13) for T1D and 1.08 (95% CI, 0.74-1.57) for T2D.

**Conclusions and Relevance:**

These findings suggest that the incidence and severity of T1D and T2D among South Korean youths increased during the COVID-19 pandemic. The cohort analysis does not support SARS-CoV-2 infection itself as being directly associated with incident diabetes.

## Key Points

**Question:**

What is the association of the COVID-19 pandemic and SARS-CoV-2 infection with youth-onset diabetes in South Korea?

**Findings:**

In this nationwide cohort of 13 639 patients younger than 20 years, new-onset type 1 diabetes and type 2 diabetes increased during the COVID-19 pandemic, with a rise in diabetic ketoacidosis at diagnosis. The risk of incident diabetes did not differ significantly between patients with and without SARS-CoV-2 infection.

**Meaning:**

These findings underscore the need for early diagnosis and timely treatment of youth-onset diabetes, and further research is required, as SARS-CoV-2 infection itself may not be directly associated.

## Teaser

This cohort study explores the incidence and severity of new-onset diabetes among youths during the COVID-19 pandemic in South Korea.

## Introduction

During the COVID-19 pandemic, an increased incidence of new-onset type 1 diabetes (T1D) among youths was reported in several countries.^1,2,3^ However, a recent cohort study in Japan revealed no increase of pediatric T1D during the pandemic.^4^ The incidence of youth-onset type 2 diabetes (T2D) escalated during the pandemic in Germany and the US.^5,6,7^

The prevalence and severity of diabetic ketoacidosis (DKA) at diagnosis of T1D in children increased during the pandemic.^3,8,9^ A multicenter study in South Korea revealed an increase in newly diagnosed pediatric T1D and T2D cases from the prepandemic period to the pandemic period, with an increased proportion of DKA at diagnosis.^10^ These studies investigated the first-year outcomes of the pandemic, but research on changes in subsequent years is scarce.

The causes behind the changes in youth-onset diabetes incidence during the pandemic remain unclear. Studies on the association of SARS-CoV-2 infection with T1D incidence have yielded conflicting results, with a multicenter study in the US reporting an elevated risk^11^ and nationwide studies in Denmark and Scotland finding no increase in risk.^2,12^ Research on the association between SARS-CoV-2 infection and incident youth-onset T2D remains lacking. Indirect factors other than SARS-CoV-2 infection during the pandemic, such as delayed access to health care and reduced physical activity due to containment measures, may have contributed to the development of diabetes.

In this study, we investigated the differences in the incidence and severity of youth-onset diabetes before and during the COVID-19 pandemic in South Korea. In addition, we explored the association between SARS-CoV-2 infection and the development of diabetes in youths.

## Methods

### Data Source

This cohort study used data produced by the Health Insurance Review & Assessment Service (HIRA) during the reimbursement of health care organizations, a process required for all health care organizations covered by health insurance in Korea. In South Korea, more than 98% of the approximately 50 million total population is covered by the National Health Insurance System (NHIS).^13^ This study was approved by the institutional review board of Seoul National University Hospital and HIRA, with a waiver of informed consent granted due to the use of anonymized data. The study followed the Strengthening the Reporting of Observational Studies in Epidemiology (STROBE) reporting guideline.

The data encompass detailed records on patient age, sex, diagnoses, type of hospital, inpatient and outpatient care, medical procedures, surgeries, tests, prescriptions, and associated costs. The HIRA data cover the same insurance claims data as the NHIS for the same population, providing a complete dataset with no missing values or loss to follow-up. Since the NHIS data are recognized as a reliable source for nationwide estimates, the Korean Diabetes Association applies operational definitions to NHIS data in the publication of the diabetes fact sheets.^14,15^

### Study Population

Patients younger than 20 years with a diagnosis of diabetes (*International Statistical Classification of Disease, Tenth Revision* [*ICD-10*] codes E10, E11, E12, E13, and E14) or COVID-19 or who underwent COVID-19 polymerase chain reaction (PCR) testing between January 1, 2016, and February 28, 2022, were included. Their claims from January 1, 2016, through February 28, 2023, were extracted from the HIRA database.

### Incident Diabetes and DKA

**Figure 1.**
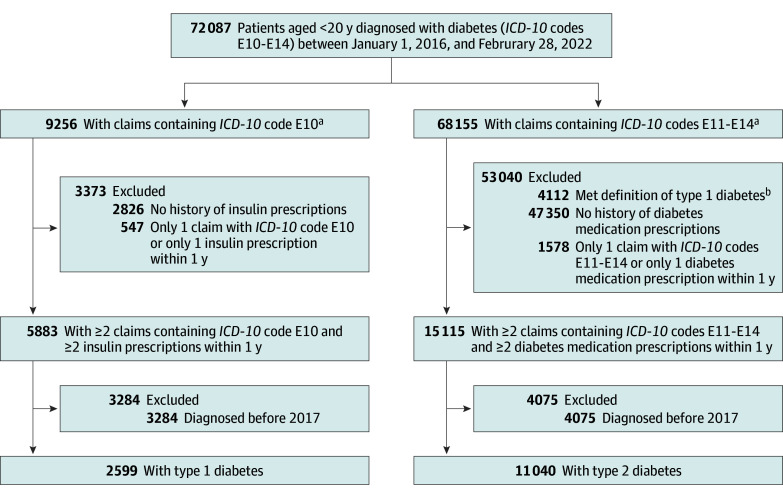
Flowchart of Patient Selection ^a^Represents the number of patients with at least 1 claim with the *International Statistical Classification, Tenth Revision* (*ICD-10*) code E10 or codes E11 to E14, with possible overlap due to patients counted in both categories. ^b^Type 1 diabetes was defined as having at least 2 claims with an *ICD-10* code E10 and at least 2 insulin prescriptions within 1 year.

We designated 2016 as the washout period and identified incident cases dated between January 1, 2017, and February 28, 2022. Incident T1D was defined as at least 2 claims with the *ICD-10* code E10 and at least 2 insulin prescriptions within 1 year.^14,16^ Incident T2D was defined as at least 2 claims with *ICD-10* codes E11 to E14 and at least 2 prescriptions of diabetes medication within 1 year.^15,17,18^ Cases with concurrent diagnoses of T1D and T2D were classified as T1D if the diagnostic criteria for T1D were met. Prescriptions for insulin or diabetes medication were included only if they were issued on separate days at least twice within 1 year. The date of diabetes onset was defined as the date of the first claims-based diabetes diagnosis. Figure 1 shows the patient selection process.

The annual incidence of diabetes was calculated using annual population data stratified by sex and age obtained from the Ministry of the Interior and Safety. An incident case of diabetes with DKA was defined as one for which the diagnosis was diabetes with coma (*ICD-10* code E1X0X) or diabetes with ketoacidosis (*ICD-10* code E1X1X) and insulin was administered within 2 weeks of those diagnoses. Among these cases, admissions to the intensive care unit (ICU) were investigated.

### Period Distinction According to the COVID-19 Pandemic

Owing to a surge in COVID-19 cases caused by the Omicron variant, South Korea has allowed the use of rapid antigen tests alongside PCR tests for diagnosis since March 14, 2022.^19^ Consequently, we defined the first pandemic year as March 1, 2020, to February 28, 2021; the second pandemic year as March 1, 2021, to February 28, 2022; and the prepandemic period as January 1, 2017, to February 29, 2020.

### Assessing the Association of SARS-CoV-2 Infection With Diabetes Risk

To evaluate the association between SARS-CoV-2 infection and the subsequent risk of new-onset diabetes, we analyzed a cohort of 1 709 206 individuals diagnosed with COVID-19 or who underwent mandatory PCR testing between March 1, 2020, and February 28, 2022, in South Korea. Data for January and February 2020 were unavailable due to insufficient anonymization. COVID-19 cases were identified based on *ICD-10* code U07.1, while negative cases were classified if individuals underwent PCR testing but were not diagnosed with COVID-19. Individuals with an initial negative result remained classified as negative for COVID-19 until the point at which a positive test result was obtained, after which they were considered positive for COVID-19 for the remainder of the study period. Individuals were followed from 30 days after the first registered PCR test until March 31, 2022, death, or first diagnosis of T1D or T2D. The first 30 days following a PCR test were excluded to minimize confounding by acute infection.^12^ Individuals with preexisting diagnoses of T1D or T2D before March 1, 2020, were excluded.

### Statistical Analysis

The data analysis was performed between January 29 and September 2, 2024. Incidence rates were calculated per 100 000 youths, categorized by sex and age group, with confidence intervals calculated using the exact Poisson method. For pairwise χ^2^ tests, *P* values were corrected using the Bonferroni method. Crude incidence rate ratios (IRRs) were estimated according to diabetes type, sex, and age group for each pandemic year compared with the prepandemic period. The adjusted IRRs were calculated using a multivariable Poisson regression model adjusted for age group (0-4 years, 5-9 years, 10-14 years, and 15-19 years) and sex. The hazard ratio (HR) for a diabetes diagnosis was estimated by comparing youths diagnosed with COVID-19 with those with negative test results for SARS-CoV-2, using a Cox regression model adjusted for sex, age group, and comorbidity (Charlson Comorbidity Index score of 0 or ≥1) and stratifying by calendar periods (quarters). A 2-sided *P* < .05 was considered statistically significant. All statistical analyses were performed using R, version 3.5.1 (R Foundation).

## Results

Between January 2017 and February 2022, 2599 youths were diagnosed with T1D (mean [SD] age, 12.0 [4.8] years; 1364 female [52.5%] and 1235 male [47.5%]), and 11 040 were diagnosed with T2D (mean [SD] age, 16.0 [2.8] years; 4179 female [37.9%] and 6861 male [62.1%]). The incidence rate of T1D increased from 5.28 per 100 000 youths (95% CI, 5.01-5.55 per 100 000 youths) before the pandemic to 6.41 per 100 000 youths (95% CI, 6.03-6.80 per 100 000 youths) during the pandemic (first pandemic year, 6.08 [95% CI, 5.57-6.60] per 100 000 youths; second pandemic year, 6.74 [95% CI, 6.19-7.29] per 100 000 youths) (Table 1). The sex- and age-adjusted IRR was 1.19 (95% CI, 1.10-1.29) during the pandemic (first pandemic year: IRR, 1.14 [95% CI, 1.03-1.26]; second pandemic year: IRR, 1.25 [95% CI, 1.13-1.38]). For T2D, the incidence rate increased from 21.18 per 100 000 youths (95% CI, 20.65-21.72 per 100 000 youths) before the pandemic to 29.28 per 100 000 youths (95% CI, 28.47-30.09 per 100 000 youths) during the pandemic (first pandemic year, 23.26 [95% CI, 22.25-24.27] per 100 000 youths; second pandemic year, 35.50 [95% CI, 34.23-36.77] per 100 000 youths). The adjusted IRR was 1.41 (95% CI, 1.36-1.46) during the pandemic (first pandemic year: IRR, 1.12 [95% CI, 1.07-1.18]; second pandemic year: IRR, 1.71 [95% CI, 1.63-1.78]).

**Table 1.** Incidence of Diabetes in Youths Younger Than 20 Years During the COVID-19 Pandemic

Period^a^	Type 1 diabetes			Type 2 diabetes		
	Events, No.	Incidence per 100 000 youths (95% CI)	IRR (95% CI)	Events, No.	Incidence per 100 000 youths (95% CI)	IRR (95% CI)
**Overall**						
Prepandemic	1495	5.28 (5.01-5.55)	1 [Reference]	5995	21.18 (20.65-21.72)	1 [Reference]
Pandemic	1104	6.41 (6.03-6.80)	1.19 (1.10-1.29)^b^	5045	29.28 (28.47-30.09)	1.41 (1.36-1.46)^b^
First year	533	6.08 (5.57-6.60)	1.14 (1.03-1.26)^b^	2038	23.26 (22.25-24.27)	1.12 (1.07-1.18)^b^
Second year	571	6.74 (6.19-7.29)	1.25 (1.13-1.38)^b^	3007	35.50 (34.23-36.77)	1.71 (1.63-1.78)^b^
**Sex**						
Male						
Prepandemic	686	4.69 (4.34-5.05)	1 [Reference]	3668	25.10 (24.29-25.91)	1 [Reference]
First pandemic year	273	6.05 (5.33-6.76)	1.29 (1.12-1.48)	1316	29.15 (27.58-30.73)	1.16 (1.09-1.24)
Second pandemic year	276	6.33 (5.58-7.08)	1.35 (1.17-1.55)	1877	43.06 (41.11-45.01)	1.72 (1.62-1.81)
Female						
Prepandemic	809	5.91 (5.50-6.32)	1 [Reference]	2327	17.00 (16.31-17.69)	1 [Reference]
First pandemic year	260	6.12 (5.38-6.86)	1.04 (0.90-1.19)	722	16.99 (15.75-18.23)	1.00 (0.92-1.09)
Second pandemic year	295	7.18 (6.36-8.00)	1.21 (1.06-1.39)	1130	27.49 (25.89-29.09)	1.62 (1.51-1.74)
**Age group, y**						
0-4						
Prepandemic	129	2.19 (1.83-2.60)	1 [Reference]	6	0.10 (0.04-0.22)	1 [Reference]
First pandemic year	48	2.86 (2.11-3.79)	1.31 (0.94-1.82)	2	0.12 (0.01-0.43)	1.17 (0.24-5.81)
Second pandemic year	53	3.46 (2.59-4.53)	1.58 (1.15-2.18)	5	0.33 (0.11-0.76)	3.21 (0.98-10.52)
5-9						
Prepandemic	270	3.85 (3.41-4.34)	1 [Reference]	114	1.63 (1.34-1.95)	1 [Reference]
First pandemic year	126	5.49 (4.58-6.54)	1.43 (1.15-1.76)	35	1.53 (1.06-2.12)	0.94 (0.64-1.37)
Second pandemic year	122	5.47 (4.54-6.53)	1.42 (1.15-1.76)	70	3.14 (2.45-3.97)	1.93 (1.43-2.60)
10-14						
Prepandemic	523	7.50 (6.87-8.17)	1 [Reference]	1359	19.48 (18.46-20.54)	1 [Reference]
First pandemic year	200	8.56 (7.41-9.83)	1.14 (0.97-1.34)	585	25.03 (23.04-27.14)	1.28 (1.17-1.42)
Second pandemic year	212	8.97 (7.8-10.26)	1.20 (1.02-1.40)	1002	42.38 (39.80-45.09)	2.18 (2.01-2.36)
15-19						
Prepandemic	573	6.81 (6.26-7.39)	1 [Reference]	4516	53.64 (52.09-55.23)	1 [Reference]
First pandemic year	159	6.47 (5.51-7.56)	0.95 (0.80-1.13)	1416	57.66 (54.70-60.74)	1.07 (1.01-1.14)
Second pandemic year	184	7.85 (6.75-9.07)	1.15 (0.98-1.36)	1930	82.30 (78.67-86.05)	1.53 (1.45-1.62)

Abbreviation: IRR, incidence rate ratio.

^a^ The prepandemic period covers January 2017 to February 2020. The first pandemic year extends from March 2020 to February 2021, and the second pandemic year extends from March 2021 to February 2022.

^b^ Calculated after adjusting for sex and age group.

**Figure 2.**
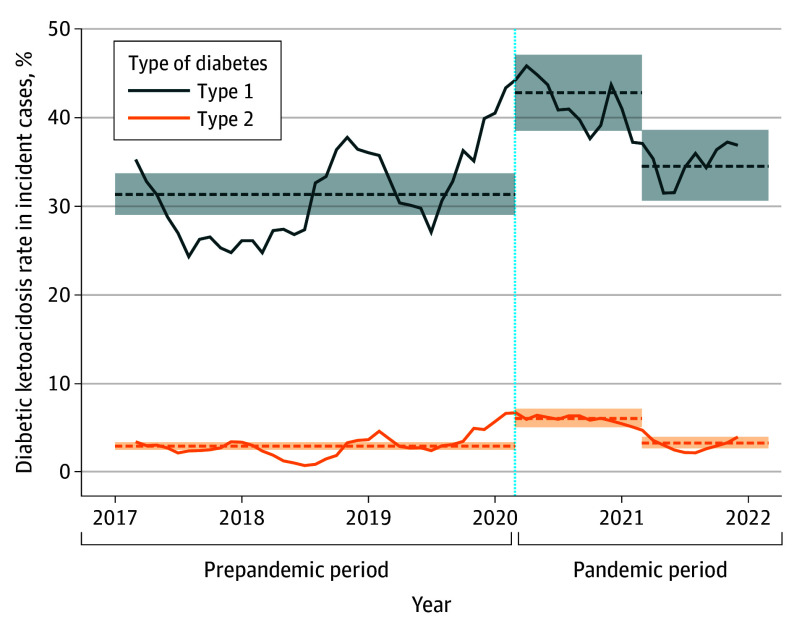
Trends in Diabetic Ketoacidosis at Diagnosis Before and During the COVID-19 Pandemic The solid line indicates the 3-month moving averages. The horizontal dashed lines and shaded areas indicate the average rates and 95% CIs for the prepandemic period, the first pandemic year, and the second pandemic year, respectively. The vertical dotted line indicates the start of the pandemic period.

The incidence of DKA at diagnosis of T1D increased from 31.3% (95% CI, 29.0%-33.7%) in the prepandemic period to 42.8% (95% CI, 38.5%-47.0%) during the first pandemic year (*P* < .001) but returned to prepandemic incidence in the second pandemic year (34.5%; 95% CI, 30.6%-38.5%; *P* = .54) (Figure 2 and Table 2). The rate of ICU admission for DKA at diagnosis of T1D increased significantly for both the first pandemic year (14.3%; 95% CI, 11.4%-17.5%; *P* < .001) and second pandemic year (13.1%; 95% CI, 10.5%-16.2%; *P* = .003) compared with the prepandemic period (8.3%; 95% CI, 7.0%-9.8%). For T2D, the incidence of DKA at diagnosis increased from 2.9% (95% CI, 2.5%-3.3%) in the prepandemic period to 6.0% (95% CI, 5.0%-7.1%) during the first pandemic year (*P* < .001), but this increase did not continue into the second pandemic year (3.2%; 95% CI, 2.6%-3.9%). The rate of ICU admission for DKA at diagnosis of T2D increased from 1.1% (95% CI, 0.9%-1.4%) in the prepandemic period to 2.5% (95% CI, 1.9%-3.3%) in the first pandemic year (*P* < .001), but returned almost to baseline (1.2%; 95% CI, 0.8%-1.6%) in the second pandemic year.

**Table 2.** DKA at Diagnosis of Diabetes in Youths During the COVID-19 Pandemic^a^

Variable	Prepandemic		First pandemic year				Second pandemic year		
	Events, No.	Rate, % (95% CI)	Events, No.	Rate, % (95% CI)	*P* value^b^	Events, No.		Rate, % (95% CI)	*P* value^b^
**Type 1 diabetes**									
No. of patients	1495	NA	533	NA	NA	571		NA	NA
DKA	468	31.3 (29.0-33.7)	228	42.8 (38.5-47.0)	<.001	197		34.5 (30.6-38.5)	.54
ICU admission for DKA	124	8.3 (7.0-9.8)	76	14.3 (11.4-17.5)	<.001	75		13.1 (10.5-16.2)	.003
**Type 2 diabetes**									
No. of patients	5995	NA	2038	NA	NA	3007		NA	NA
DKA	172	2.9 (2.5-3.3)	122	6.0 (5.0-7.1)	<.001	97		3.2 (2.6-3.9)	>.99
ICU admission for DKA	68	1.1 (0.9-1.4)	51	2.5 (1.9-3.3)	<.001	35		1.2 (0.8-1.6)	>.99

Abbreviations: DKA, diabetic ketoacidosis; ICU, intensive care unit; NA, not applicable.

^a^ The prepandemic period covers January 2017 to February 2020. The first pandemic year extends from March 2020 to February 2021, and the second pandemic year extends from March 2021 to February 2022.

^b^ The *P* value compares each pandemic year with the prepandemic period as the reference.

Among the 1 706 483 youths (804 577 person-years) who underwent SARS-CoV-2 PCR testing, 470 929 (27.6%) were diagnosed with COVID-19 at least once. During the follow-up period, 115 and 447 youths developed T1D and T2D, respectively (Table 3). The HR for developing T1D following SARS-CoV-2 infection was 0.44 (95% CI, 0.17-1.13; *P* = .09) and for T2D, 1.08 (95% CI, 0.74-1.57; *P* = .69).

**Table 3.** Hazard Ratios for New-Onset Diabetes According to SARS-CoV-2 Infection in Youths

Variable	SARS-CoV-2 positive			SARS-CoV-2 negative			AHR (95% CI)^a^	*P* value
	Events, No.	At risk, No.	Follow-up, person-y	Events, No.	At risk, No.	Follow-up, person-y		
Type 1 diabetes	5	470 896	64 034	110	1 296 016	740 294	0.44 (0.17-1.13)	.09
Type 2 diabetes	33	470 924	64 043	414	1 296 320	740 489	1.08 (0.74-1.57)	.69

Abbreviation: AHR, adjusted hazard ratio.

^a^ The AHR is calculated considering the time-dependent exposure to SARS-CoV-2 status and is adjusted for age group, sex, and comorbidity (Charlson Comorbidity Index score of 0 or ≥1) and stratified by calendar periods (quarters).

## Discussion

In this cohort study, we found that the incidence of T1D and T2D increased among youths during the COVID-19 pandemic in South Korea. The incidence of DKA at diagnosis of diabetes increased in the first pandemic year compared with the prepandemic period but not during the second pandemic year. The risk of new-onset diabetes did not increase with SARS-CoV-2 infection.

We used claims data without laboratory results for autoantibodies, which are crucial for discriminating T1D. South Korea’s detailed classification system requires patients to register with the National Registration for Diabetes to receive reimbursement for consumable diabetes-related supplies.^20^ This system includes specific criteria, such as low C-peptide levels, a history of DKA, or positive test results for autoantibodies, along with *ICD-10* code E10 and insulin prescription to classify a patient as having T1D.^21^ This comprehensive system helps minimize misclassification. Validation studies using claims data have shown a high positive predictive value of 94% to 96.3% across various countries.^22,23^ The operational definition that combines diagnosis codes and diabetes medication prescriptions within NHIS claims data in Korean adults with T2D has shown comparable accuracy and specificity when validated against a nationally representative sample cohort with laboratory information.^24^ Nonetheless, further validation studies are necessary to confirm its accuracy within the Korean health care setting.

The incidence of T1D varies widely, with lower rates observed in East Asian compared with Western populations.^25^ A meta-analysis showed T1D incidence ranging from 5.88 to 37.60 per 100 000 person-years before the pandemic and 5.88 to 40.20 after the pandemic.^26^ Only 1 Asian study, from Oita Prefecture in Japan, was included, with a T1D incidence of 5.78 per 100 000 person-years before the pandemic and 5.92 per 100 000 person-years during the pandemic, similar to our T1D incidence.^4^ Most studies on youth-onset diabetes focused primarily on T1D, leading to a lack of data on T2D. A recent systematic review reported T2D incidence in the top 10 countries with the highest incidence, ranging from 65 to 734 per 100 000 persons.^27^ The T2D incidence in our study was higher than the 5.6 per 100 000 persons from 2010 to 2013 in Jeonbuk Province, South Korea, but a direct comparison is limited by differences in study period and regional data.^28^

The increase in the incidence of T1D among Korean youths during the COVID-19 pandemic is consistent with findings from a multicenter study in South Korea.^10^ This trend also aligns with nationwide studies conducted in Germany, Denmark, and Finland.^1,3,29^ However, other studies have shown conflicting results. A French study indicated no increase in hospitalizations for new-onset T1D during the pandemic.^30^ Moreover, a web-based survey in Italy revealed a decrease in the incidence in 2020.^31^ Meanwhile, a study from Oita Prefecture in Japan revealed an annual increase of 4.7% from 1999 to 2021 without an additional rise during the pandemic.^4^ Notably, the studies reporting no increase in T1D incidence used relatively limited definitions, such as a single diagnosis of T1D, or were not nationwide in scope. In contrast, several comprehensive nationwide studies, including our study, consistently showed an increase in T1D incidence during the pandemic.^1,3,29^

Although research on the incidence of youth-onset T2D during the pandemic is lacking, the observed increase in the incidence is in line with reports from other countries. A nationwide study of German children and adolescents revealed an increased incidence of T2D during the pandemic.^5^ Similarly, multicenter and single-center studies from the US have revealed an increase in the incidence of T2D among youths during pandemic.^6,7,32^

The incidence of DKA in youths significantly increased for both T1D and T2D during the first pandemic year compared with the prepandemic period. Consistent with our findings, studies from Finland, Germany, and Italy have revealed increases in the incidence of DKA at the diagnosis of T1D during the pandemic.^3,33,34^ Other studies from the US and South Korea observed an increasing trend in the incidence of DKA for T2D, although these studies were not based on nationwide data.^6,10,35^ The increase in DKA may be associated with delays in the diagnosis and treatment of diabetes. Studies from Finland and Germany revealed elevated hemoglobin A_1c_ levels at diagnosis during the pandemic, suggesting delays in diagnosis.^3,36^ Furthermore, a multinational study showed a correlation between the stringency index—which reflects the severity of pandemic-related containments, such as school closures and limitations on group gatherings—and the increased incidence of DKA.^8^ Restricted hospital access due to pandemic containment measures, as indicated by a 50.4% reduction in pediatric emergency department visits in South Korea in 2020 compared with 2018-2019, may have contributed to the increase in DKA cases.^37^ The return to prepandemic DKA rates during the second pandemic year in our study may have been influenced by faster emergency interventions that initiated in late 2020. These interventions included the introduction of rapid diagnostic tests, such as emergency screening PCR kits and rapid antigen tests, despite unchanged social distancing measures.^38,39^ However, we observed that the rate of ICU admissions for DKA in T1D consistently increased during both the first and second pandemic years, emphasizing the necessity of special attention for patients with new-onset T1D, particularly severe cases, during future periods of disease containment measures.

Our findings did not confirm an association between SARS-CoV-2 infection and new-onset T1D and T2D. The relatively low incidence of diabetes in South Korea, along with the surge in SARS-CoV-2 infections among youths during the later part of our study period, resulted in a limited sample size and constrained our ability to draw definitive conclusions. Two national registry studies from Denmark and Scotland reported no substantial increase in the risk of T1D 30 days after a positive SARS-CoV-2 test result.^1,2,12^ Similarly, Finland’s national registry study indicated a low presence of SARS-CoV-2 antibodies in children with new-onset T1D during the pandemic, suggesting minimal direct linkage between SARS-CoV-2 infection and T1D.^3^ Multinational cohort studies also found no association between prior SARS-CoV-2 infection and the development of autoimmunity related to T1D.^40,41^ However, a meta-analysis including both adults and children reported that SARS-CoV-2 infection was associated with an increased risk for T1D and T2D.^42^ In a multicenter study spanning 15 countries and the US, data from 2 large databases showed an elevated risk of newly diagnosed pediatric diabetes following SARS-CoV-2 infection compared with other respiratory viruses.^11,43^ The generalizability of these studies is limited by the inclusion of an adult population or the use of non–nationally representative data. While adult studies have suggested that SARS-CoV-2 infection may increase the risk of T2D as part of post–COVID-19 condition, there is a lack of research on the association between SARS-CoV-2 infection and T2D in youths.^44,45^

The increase in youth-onset diabetes during the pandemic may be possibly attributable to the socioenvironmental changes associated with the COVID-19 pandemic. Social distancing and heightened hygiene measures during the pandemic may have not only reduced the risk of SARS-CoV-2 infection but also limited healthy microbial exposure, decreasing microbial diversity.^46^ This reduction may trigger autoimmune diseases such as T1D by increasing host inflammation.^46,47^ Additionally, concerns about SARS-CoV-2 and school closures led to increased social isolation, depression, and anxiety among youths, which may have negatively affected mental health and potentially contributed to the rise in both T1D and T2D.^48,49,50,51^ Furthermore, increased screen time, decreased physical activity, and dietary shifts toward ultraprocessed, calorie-dense foods may have contributed to the global rise in obesity, influencing the higher incidence of T2D.^49,52,53^

### Strengths and Limitations

The main strength of this study is its use of nationwide data on the incidence of diabetes among Korean youths before and during the pandemic period. Moreover, this study evaluated prolonged effects of the pandemic up to the second year, whereas most previous studies focused only on the first year.

The study also has several limitations. First, we relied on claims data, which lack key biochemical markers and clinical details, such as autoantibodies and obesity status, potentially influencing the accuracy of diabetes incidence determinations. Second, the use of stringent criteria may have resulted in underdiagnosis, particularly of T2D. However, the likelihood of undiagnosed diabetes is low in South Korea due to routine student health screenings.^28^ Third, we selected an age cutoff of 20 years as the incidence of T2D is higher in late adolescence. We also divided the age groups into 5-year intervals to facilitate more precise comparisons of incidence rates. Finally, due to the low incidence of youth-onset diabetes, the statistical power to analyze the association between SARS-CoV-2 infection and subsequent diabetes risk may be limited, highlighting the need for further studies in larger populations.

## Conclusions

In this cohort study, we observed an increased incidence and severity of both T1D and T2D among youths during the COVID-19 pandemic (March 2020 to February 2022) compared with the prepandemic period (January 2017 to February 2020) in South Korea. Rates of DKA increased in the first pandemic year but returned to prepandemic levels during the second year. Diabetes risk was not predisposed among youths with SARS-CoV-2 infection. Further studies are needed to thoroughly analyze the impact of pandemic-related socioenvironmental changes on the risk of developing youth-onset diabetes.

## Notes

Supplement 1.
Data Sharing Statement
