# Supplementary material for: New-Onset Type 1 and Type 2 Diabetes Among Korean Youths During the COVID-19 Pandemic
Source: JAMA Pediatr. 2024 Dec 9;179(2):155–62. doi: 10.1001/jamapediatrics.2024.5068 (PMC11791714; doi:10.1001/jamapediatrics.2024.5068)
Supplement: Supplement 1. — Data Sharing Statement [file jamapediatr-e245068-s001.pdf]

Data Sharing Statement

Lee. New-Onset Type 1 and Type 2 Diabetes Among Korean Youths During the COVID-19 Pandemic. *JAMA Pediatr*. Published December 09, 2024.
doi:10.1001/jamapediatrics.2024.5068

Data
Data available: No

Additional Information
Explanation for why data not available: The data that support the findings of this study are available from Health Insurance Review & Assessment Service, but restrictions apply to the availability of these data, which were used under license for the current study and therefore are not publicly available.
